# Self-assemble nanoparticles based on polypeptides containing C-terminal luminescent Pt-cysteine complex

**DOI:** 10.1038/srep41991

**Published:** 2017-02-03

**Authors:** E. G. Vlakh, E. V. Grachova, D. D. Zhukovsky, A. V. Hubina, A. S. Mikhailova, J. R. Shakirova, V. V. Sharoyko, S. P. Tunik, T. B. Tennikova

**Affiliations:** 1 Saint-Petersburg State University, Institute of Chemistry, Universitetsky pr. 26, 198504 St. Petersburg, Russia

## Abstract

The growing attention to the luminescent nanocarriers is strongly stimulated by their potential application as drug delivery systems and by the necessity to monitor their distribution in cells and tissues. In this communication we report on the synthesis of amphiphilic polypeptides bearing C-terminal phosphorescent label together with preparation of nanoparticles using the polypeptides obtained. The approach suggested is based on a unique and highly technological process where the new phosphorescent Pt-cysteine complex serves as initiator of the ring-opening polymerization of α-amino acid N-carboxyanhydrides to obtain the polypeptides bearing intact the platinum chromophore covalently bound to the polymer chain. It was established that the luminescent label retains unchanged its emission characteristics not only in the polypeptides but also in more complicated nanoaggregates such as the polymer derived amphiphilic block-copolymers and self-assembled nanoparticles. The phosphorescent nanoparticles display no cytotoxicity and hemolytic activity in the tested range of concentrations and easily internalize into living cells that makes possible *in vivo* cell visualization, including prospective application in time resolved imaging and drug delivery monitoring.

Bioimaging based on luminescent microscopy represents one of the most powerful analytical techniques in the life sciences because of its high sensitivity accompanied with simplicity and low cost. This visualization procedure can be carried out using water soluble organic and organometallic dyes, their conjugates with polymers and biomolecules^1^,^2^,^3^, as well as with luminescent nanoobjects^4^,^5^,^6^,^7^. The growing attention to the latter approach can be explained by well known practice of application of the nanoparticles to construct advanced drug delivery systems, which also make possible easy visualization of drug distribution in cells and tissues. During the last years, such smart combination of diagnostic and therapeutic properties caused enormous interest in biomedical research area.

Nowadays, the nanocarriers intended for a creation of drug delivery systems can be both of inorganic^8^ and organic nature^9^,^10^. Among inorganic nanoparticles applied for bioimaging and drug delivery such systems as dye-doped silica^11^, quantum dots^12^, metal nanoclusters^13^, lanthanide-doped nanoparticles^14^, etc. have got a particular attention. The luminescent properties of organic nanocarriers are usually associated with the native material emission characteristics or are the result of their labeling with emissive moieties. In particular, the materials based on photo-luminescent polyacrylonitrile can be mentioned as an example of label-free organic nanoparticles for bioimaging^15^. However, both the covalent labeling of organic nanoparticles or encapsulation of a dye inside the particles^16^,^17^ are the most common approaches. Encapsulation of dyes in a drug-carrying nanoparticle is usually aimed at synchronous release of drug and dye to signal about drug availability in biological system. Such a process can occur due to the biodegradation of nanoparticles^18^, or is a result of nanocarrier response to external stimuli (temperature, pH, or some others)^19^. Another way of monitoring of drug-carrier localization is preparation of nanoparticles containing covalently bound dye molecules, which represent a stable form of luminescent nanoobjects. Currently, there are well elaborated approaches to prepare functionalized luminophores^19^ and ways of their covalent binding to nanocontainers^20^,^21^, which however needs a special chemical procedures to link the probe to a certain chemical function of the nanocontainer like for example conjugation of polymer material functional groups with the reactive moiety of the dye to give the luminescent nanoobjects^22^. This approach normally gives an even distribution of labels on the surface of nanocarrier. In contrast, modification of polymer end-functional group allows for preparation of uniformed nanoparticles with strictly defined localization of luminescent molecule^23^.

One of the prospective groups of nanoparticles, which are widely considered as drug delivery systems^24^,^25^,^26^, as well as materials for the realization of two-photon fluorescence bioimaging^27^,^28^, is so called *soft materials.* These objects mostly represent self-assembled nanostructures obtained from amphiphilic precursors, in which the hydrophobic part is responsible for self-aggregation in aqueous media, whereas hydrophilic segment is responsible for both solubility and chemical functionality.

In the present communication we report on a novel approach to the synthesis of amphiphilic polypeptides bearing C-terminal phosphorescent label followed by preparation of luminescent nanoparticles. Taking into account the biodegradability, biocompatibility and wide variability of suitable functional groups, the amphiphilic polypeptides are very attractive candidates for preparation of nanoparticles of different morphology targeted to the biomedical applications^29^,^30^,^31^. The synthesis of amphiphilic co-polypeptides was carried out using ring-opening polymerization strategy (ROP) of N-carboxyanhydrides (NCAs) of α-amino acids. Traditionally, such polymerization is initiated with amines, but only primary amines provide narrow molecular weight distribution of resulting polymer product^32^,^33^. In our research we suggested an original approach based on the use of NH_2_-bearing luminescent organometallic complex as initiator for NCA polymerization. In particular, a luminescent Pt-cysteine complex with emission in green area of visible spectrum was specially designed and applied as polymerization initiator. The advantages of the application of phosphorescent organometallic probes over fluorescent organic dyes are higher photostability of the complexes and higher sensitivity of time resolved imaging experiments due to the opportunity to cut off background emission in time-gated regime^34^. Moreover, the diversity of the complexes properties determined by easy variation of their ligand environment and the nature of the metal center allows for the rational choice of the complexes with appropriate absorption-emission characteristics^35^,^36^,^37^,^38^.

The developed approach made possible attachment of the luminescent label at C-terminal position of the synthesized polypeptide that strictly determined location of the label on the nanoparticle outer (hydrophilic) surface after amphiphilic copolymer self-assembling. This design of the nanocarrier is extremely important to increase brightness of the probe and consequently sensitivity of the imaging experiments.

## Experimental Section

### Materials

*L*-cysteine, γ-benzyl-L-glutamate ((Bzl)Glu), ɛ-Z-L-lysine ((Z)Lys), *L*-leucine (Leu), triphosgene, α-pinene, trifluoromethanesulfonic acid (TFMSA), trifluoroacetic acid (TFA), 2-(p-tolyl)-pyridine (N^C^tpy) and other reagents were purchased from Sigma–Aldrich (Germany) and used as received. 1,4-Dioxane, n-hexane were purchased from Vecton Ltd. (Russia) and distilled prior to use. Dimethyl sulfoxide (DMSO), also purchased from Vecton Ltd. (Russia), was dried under molecular sieves 4 Ǻ and distilled under vacuum. Ethyl acetate purchased from Panreac (Spain) was distilled before application. All solvents were purified and distilled using standard procedures.

HEK 293 (human embryonic kidney cells), HELF (human embryonic lung fibroblasts) and U937 (human lymphoblast lung cells) cell lines were purchased from BioloT (Russia). The cells were cultured in Dulbecco’s Modified Eagle Medium (BioloT, Russia) and grown in Dulbecco’s Modified Eagle’s Medium containing 10% (v/v) heat-inactivated fetal bovine serum (FBS, HyClone Laboratories, UT, USA), 1% L-glutamine, 1% sodium pyruvate, 50 U/mL penicillin and 50 μg/mL streptomycin (BioloT).

### Instruments

The solution ^1^H NMR spectra were recorded on a Bruker Avance 400 spectrometer. Mass spectra were recorded on a Bruker microTOF 10223 instrument at ESI^+^ mode (solvent – MeOH). UV/Vis absorption and emission spectra were recorded with a Shimadzu UV-1800 spectrophotometer (Japan), a HORIBA Scientific FluoroLog-3 spectrofluorometer and Avantes spectrometer. Gel-permeation chromatography (GPC) was performed using Shimadzu LC-20 Prominence system equipped with refractometric RID 10-A detector (Japan) and 7.8 × 300 mm Styragel Column HMW 6E, 15–20 μm bead size (Waters, USA). The GPC calculations were fulfilled with GPC LC Solutions software (Shimadzu, Japan). Amino acid HPLC analysis was carried out using an LCMS-8030 Shimadzu system with Mass-detection (LC-MS) (Shimadzu, Japan), equipped with 2 × 150 mm Luna C_18_ column, packed with 5 μm particles. Dynamic light scattering (DLS) and zeta potential measurements of colloids were performed using a Zetasizer ZS device (Malvern, Great Britain) at angle 113°.

### Synthesis of *(cis)*-[Pt(N^C^tpy)(1,3-dibenzylbenzimidazol-2-yliden)Cl] (1)

The platinum(II) complex [Pt(N^C^tpy)(DMSO)Cl] and 1,3-dibenzyl-1H-benzo[d]imidazol-3-ium chloride were prepared according to the published procedures^39^,^40^. To synthesize **1** the compound [Pt(N^C^tpy)(DMSO)Cl] (200 mg, 0.418 mmol), 1,3-dibenzylbenzimidzolium chloride (154 mg, 0.460 mmol) and K_2_CO_3_ (288 mg, 2.09 mmol) were added to 40 ml of acetone and the mixture was stirred overnight at 40 °C. The resulting mixture was evaporated to dryness, redissolved in dichloromethane, filtered to remove potassium salts and evaporated to dryness again. The crude residue was purified by flash column chromatography (Silica, eluent – dichloromethane) to afford two fractions which were evaporated to give **1** (123 mg, 43%) and its *trans*-isomer 1^*^ (147 mg, 51%) as yellow solids.

1^*^:^ 1^Н NMR (acetone-d_6_, ambient temperature): δ (ppm) 8.12 (d with broad ^195^Pt satellites, ^3^*J*_HH_ = 6.4 Hz, 1H, H_1_), 8.00 (s with broad ^195^Pt satellites, 1H, H_8_), 7.94 (m, 1H, H_3_), 7.87 (m, 1H, H_4_), 7.66 (m, 4H, H_12_), 7.55–7.51 (m, 3H, H_5_ and H_10_), 7.30–7.28 (m, 2H, H_11_), 7.25−7.19 (m, 6H, H_13_ and H_14_), 6.92 (d, ^3^*J*_HH_ = 7.9 Hz, 1H, H_6_), 6.83 (m, 1H, H_2_), 6.23 (d, ^2^*J*_HH_ = 15.2 Hz, 2H, H_9_), 6.10 (d, ^2^*J*_HH_ = 15.2 Hz, 2H, H_9_’), 2.34 (s, 3H, H_7_). Proton numbering scheme is given in Fig. S1.

**1:** ^1^Н NMR (acetone-d_6_, ambient temperature): δ (ppm) 9.63 (d with broad ^195^Pt satellites, ^3^*J*_HH_ = 5.3 Hz, 1H, H_1_), 8.04 (m, 1H, H_3_), 7.98 (m, 1H, H_4_), 7.73 (m, 4H, H_12_), 7.61 (d, ^3^*J*_HH_ = 7.7 Hz, 1H, H_5_), 7.43 (m, 3H, H_2_ and H_10_), 7.30−7.22 (m, 8H, H_11_, H_13_ and H_14_), 6.92 (d, ^3^*J*_HH_ = 7.7 Hz, 1H, H_6_), 6.63 (m, ^3^*J*_H-Pt_ = 34.6 Hz, 1H, H_8_), 6.22 (d, ^2^*J*_HH_ = 15.5 Hz, 2H, H_9_), 6.10 (d, ^2^*J*_HH_ = 15.5 Hz, 2H, H_9_’), 2.13 (s, 3H, H_7_). Proton numbering scheme is given in Fig. S2. ESI + (*m/z*): [M − Cl]^+^ 661.20, [M + K]^+^ 735.32, [M + K + acetone]^+^ 793.37.

### Synthesis of *(cis)*-[Pt(N^C^tpy)(1,3-dibenzylbenzimidazol-2-yliden)(*L*-cysteine)] (2)

The complex **1** (17 mg, 0.0244 mmol) and *L*-cysteine (9 mg, 0.0742 mmol) were dissolved in 4 ml of the dichloromethane/methanol (1:1 v/v) mixture. Triethylamine (30 μl, 21.9 mg, 0.216 mmol) was added *via* syringe and the mixture was stirred overnight at room temperature. The reaction completion was monitored by the ^1^H NMR referenced to *L*-cysteine resonances. The resulting solution was evaporated to dryness. The residue washed with 2 × 3 ml of dichloromethane, 3 × 5 ml of water and dried in nitrogen stream. The solid obtained was dissolved in 5 ml of methanol, filtered to remove insoluble impurities and the solution was evaporated to give **2** as yellow solid (10.3 mg, 54%)^1^.Н NMR (methanol-d_4_, ambient temperature): δ (ppm) 9.27 (d with broad ^195^Pt satellites, ^3^*J*_HH_ = 5.3 Hz, 1H, H_1_), 7.93 (m, 1H, H_3_), 7.87 (m, 1H, H_4_), 7.54 (m, 5H, H_12_ and H_5_), 7.44 (m, 2H, H_10_), 7.24 (m, 9H, H_2_, H_11_, H_13_ and H_14_), 6.86 (d, ^3^*J*_HH_ = 7.2 Hz, 1H, H_6_), 6.37 (t, ^3^*J*_HPt_ = 28.9 Hz, 1H, H_8_), 6.30 (d, ^2^*J*_HH_ = 15.4 Hz, 1H, H_9_), 6.29 (d, ^2^*J*_HH_ = 15.3 Hz, 1H, H_9_*), 5.90 (d, ^2^*J*_HH_ = 15.3 Hz, 1H, H_9_’), 5.86 (d, ^2^*J*_HH_ = 15.4 Hz, 1H, H_9_*’), 3.47 (m, 1H, H_15_), 2.72 (m, 1H, H_16_), 2.58 (m, 1H, H_16_’), 2.04 (s, 3H, H_7_). Proton numbering scheme is given in Fig. S3. ESI + (*m/z*): C_33_H_28_N_3_Pt [M − Cys]^+^ 661.19.

### Synthesis of N-carboxyanhydrides (NCA) of α-amino acids

NCA monomers of γ-benzyl-L-glutamate, ɛ-Z-L-lysine, L-leucine, were prepared as described elsewhere^41^. The structure and purity of NCAs obtained was proved by ^1^H NMR at 25 °C in СDCl_3_. ^1^Н NMR (methanol-d_4_, ambient temperature): (**Bzl)Glu NCA**: δ 7.44–7.30 (m, 5H), 6.43 (s, 1H), 5.14 (s, 2H), 4.37 (t, *J* = 6.0 Hz, 1H), 2.60 (t, *J* = 6.8 Hz, 2H), 2.29 (m, 1H), 2.12 (m, 1H); (**Z)Lys NCA**: δ 7.44–7.28 (m, 5H), 6.61 (s, 1H), 5.26–5.01 (m, 2H), 4.88 (s, 1H), 4.27 (s, 1H), 3.27–3.16 (m, 2H), 1.97 (m, 1H), 1.83 (m, 1H), 1.73–1.28 (m, 4H); **Leu NCA**: δ 7.14 (s, 1H), 4.35 (dd, J = 8.8, 3.6 Hz, 1H), 1.96–1.75 (m, 2H), 1.72–1.66 (м, 1H), 0.98 (dd, J = 8.3, 6.1 Hz, 6H).

### Synthesis and characterization of amphiphilic polypeptides labeled with 2

P(Z)Lys and P(Bzl)Glu were synthesized by ROP of appropriate NCAs using complex **2** as initiator. The following amounts of **2** (0.015 g) and NCAs (0.26 g for (Bzl)Glu and 0.60 g for (Z)Lys) were dissolved in dried DMSO (4 wt%) and the solution was incubated for reaction to proceed at 35 °C for 48 h. The product was precipitated with an excess of diethyl ether. To remove oligomers and unreacted low molecular weight compounds, the precipitate was washed with CHCl_3_. Molecular-weight characteristics of acquired **2**-P(Z)Lys and **2**-P(Bzl)Glu were determined *via* GPC method. The analysis was performed at 60 °C using 0.1 M LiBr solution in DMF as eluent. The mobile phase flow rate was 0.3 mL/min. Molecular weights and molecular weight distributions for homopolymers were calculated using poly(methyl methacrylate) standards in *M*_*w*_ range from 17,000 to 250,000 with polydispersity lower than 1.14.

Labeled homopolymers **2**-P(Z)Lys and **2**-P(Bzl)Glu were applied as macroinitiators for polymerization of Leu. Solutions of NCA (1 wt%) in DMSO containing 0.17 g Leu NCA and 9.5 μmol of **2**-P(Z)Lys or 0.13 g Leu NCA and 15 μmol of **2**-P(Bzl)Glu were incubated at 35 °C for 48 h. The copolymers were precipitated and purified as described above for homopolymers. The hydrophobic block contribution was evaluated by amino acid analysis using reversed-phase HPLC. The polypeptide samples were initially hydrolyzed as follows: 1 mg of a sample was dissolved in 2 mL of 6 M HCl containing 0.0001% phenol. The mixture was heated in vacuum-sealed tube for 4 days at 110 °C. Then the reaction mixture was diluted with distilled water and evaporated *in vacuo* to remove HCl. For HPLC analysis, the isocratic elution mode was applied and 0.1% acetonitrile/НСООН in a ratio 5/95 wt% was used as eluent. The mobile phase flow rate was equal to 0.3 mL/min, sample volume 10 μL.

### Nanoparticles preparation and characterization

The Bzl- and Z-protective groups of copolymers were removed by addition of 0.042 mL TFMSA and 0.028 mL of TFA to 0.07 g of copolymer in 2.8 mL of DMSO and further incubation of the reaction mixture at 22 °C for 5 hours. After removing protective groups, the block copolymer was purified *via* dialysis (MWCO 1000) against DMSO for 12 h. Polymer nanoparticles were prepared by solvent inversion during dialysis (MWCO 1000) against 0.1 M PBS (pH 7.4) during 24 h, followed by freeze drying and final dispersing for 2 hours under sonication in chosen buffer at concentration of 0.5 mg/mL. The colloids obtained were then characterized by DLS method for measurement of hydrodynamic diameter of the prepared nanoobjects. TEM images was carried out as described in literature^31^.

### Cell experiments

Cytotoxicity assay of prepared particles was performed using the same protocol as described elsewhere^42^. The incubation time was 24 h.

Blood samples from healthy volunteer were collected from cubital vein into vacutainers containing 124 mmol/L sodium citrate. Separation of erythrocytes was performed by centrifugation at 2000× g for 5 min. Pellets of erythrocytes were washed 5 times with isotonic sodium chloride solution. After washing, the collected erythrocytes were resuspended in isotonic sodium chloride solution and test substances were added. The reference sample was prepared by suspending of erythrocytes in 250 μL deionized water. Aliquots 250 μL of polypeptide particles of different concentrations were added to 250 μL of resuspended erythrocyte colloids. The samples were incubated for 1 h at 37 °C in CO_2_-incubator with gentle shaking. At the end of incubation period, centrifugation was done for 5 min at 2000× g. The aliquots of 200 μL supernatants were transferred into 96-well transparent plate and analyzed spectrophotometrically at 415 nm. The percentage of hemolysis induced by deionized water was taken as 100%.

For visualization experiment, the U937 cell line was used. 500 μl containing 2 × 10^5^ cells were placed in a glass chamber (LabTec-II with CC2 treatment) and cultured at 37 °C in the CO_2_-incubator for 24 h. At the end of incubation period, the medium was exchanged with a fresh medium containing 0.5 mg/mL of polypeptide nanoparticles and samples were incubated for 1 h at 37 °C in the CO_2_-incubator. The cells were then washed with pre-warmed PBS buffer three times and 100μl of U937 cell suspension was pipetted on a microscope slide. A coverslip was placed over the object for viewing by scanning confocal microscope. The images were acquired at the excitation wavelengths varying from 375–400 nm and emission wavelengths were scanned from 500–530 nm, respectively.

## Results and Discussion

### Synthesis and characterization of Pt-cysteine luminescent complex

**Figure 1:**
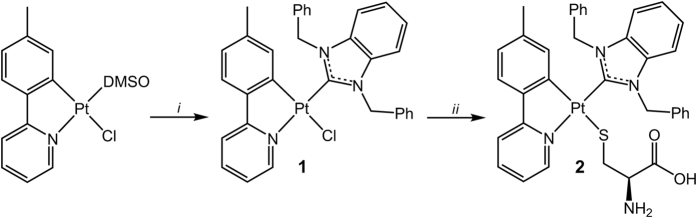
Synthesis of the complexes **1** and **2** (***i*** **–** 1,3-dibenzylbenzimidzolium chloride, K_2_CO_3_, acetone, 40 °C; ***ii*** – *L*-cysteine, methanol/dichloromethane mixture, triethylamine, 22 °C).

Complex **1** was synthesized by the reaction of [Pt(N^C^tpy)(DMSO)Cl] with 1,3-dibenzylbenzimidzolium chloride in a presence of K_2_CO_3_ (Fig. 1)^39^. The reaction results in formation of *cis*- and *trans*-isomers, which were chromatographically separated. The *cis*-isomer was chosen for the further studies because it displays higher emission quantum yield. Compound **2** was obtained by the reaction of **1** with *L*-cysteine in the presence of triethylamine (Fig. 1).

The compounds **1** and **2** were characterized using the ^1^H NMR spectroscopy and ESI^+^ mass-spectrometry. The ^1^H NMR spectra of the complexes fit completely the structural patterns shown in Fig. 1. The low field part of the both **1** and **2** ^1^H NMR spectra (from 9.27 to 5.86 ppm) displays a clearly resolved set of the signals corresponding to the cyclometallated and carbene ligands (see Figs S2 and S3, Supporting Information). In the spectrum of **2** the resonances in range from 3.49 to 2.35 ppm are assigned to S-coordinated deprotonated *L*-cysteine moiety. The ESI^+^ mass spectra of **1** and **2** contain the signal corresponding to the monocations [M−Cl]^+^ and [M−Cys]^+^. respectively (see Figs S4 and S5 of Supporting Information). The latter signal appears because the coordinated *L*-cysteine fragment can be easily protonated followed by dissociation from the metal center under the conditions of ESI^+^ experiment.

**Figure 2:**
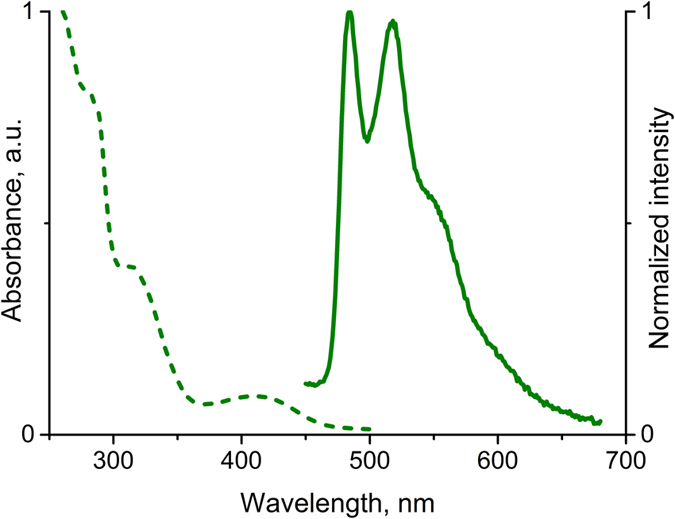
Absorption (CH_2_Cl_2_, dash line) and normalized emission (acetone, λ_exc_ = 380 nm, ambient temperature, solid line) spectra of 2.

The complex **2** is expectedly luminescent in solution, absorption and emission spectra are shown in Fig. 2, spectroscopic parameters and photophysical data obtained are summarized in Table 1. In solution **2** displays an intense high-energy absorption in the 260–350 nm interval, which can be assigned to intraligand electronic transitions, whereas relatively weak absorption bands in the 360–450 nm range originate from MLCT transitions typical of cyclometallated Pt^II^ complexes^43^,^44^,^45^,^46^. Excitation of **2** at 380 nm both in acetone or dichloromethane gives typical green emission (Fig. 2) with quantum yield of ca. 6%. Large Stokes shift together with the lifetime value of 0.29 μs in degassed solution are indicative of triplet nature of the emission, i.e. phosphorescence. In the aerated solutions, the emission intensity decreases appreciably that clearly points to the effective luminescence quenching by molecular oxygen and provides further support to the triplet nature of emission. The data obtained for methanol solution (Table 1) indicates that this solvent effectively quenches the luminescence of compound **2**. The emission bands of **2** display clearly resolved vibrational structure with the spacing of ca. 1370 cm^−1^ that fits well with the vibrational frequencies of phenylpyridine moiety and clearly points to the strong involvement of the cyclometallated ligand orbitals into emissive excited state^47^. According to the previous studies this emission is determined by the mixed ^3^IL(π→π^*^)/^3^MLCT(dπ→π^*^) transitions with a prevalence of the former^43^,^44^,^45^,^46^.

### Synthesis and characterization of labelled amphiphilic polypeptides

**Figure 3:**
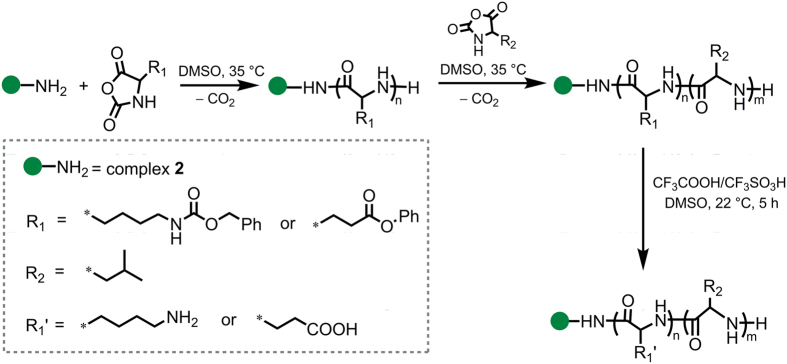
Synthesis of **2**-bearing amphiphilic copolypeptides.

The synthesized luminescent compound **2** containing primary amino group was used as an initiator of ROP for both γ-(Bzl)Glu and ε-(Z)Lys NCAs. According to the known mechanism of NCA polymerization initiated by primary amines the reaction resulted in attachment of the initiator molecule to C-terminal position of the polypeptide chain^32^. The homopolypeptide obtained, also contains terminal NH_2_-group and can be applied as a macroinitiator for the synthesis of block-copolymer. The suggested strategy of the synthesis of amphiphilic polypeptides bearing C-terminal luminescent dye is illustrated in Fig. 3.

In principle the polymer chain bound to the phosphorescent chromophore may affect the luminescent properties of the label to give a partial or even a complete emission quenching depending on the length of the polymer. To evaluate this effect in the conjugates obtained, the conditions of synthesis were varied to prepare the polypeptides of different length. In compliance with the discussed step-wise synthesis, two amphiphilic polypeptide conjugates, **2**-PLys_n_-*b*-PLeu_m_ and **2**-PGlu_n_-*b*-PLeu_m_, were synthesized. In these macromolecular products, PGlu and PLys are hydrophilic polymers containing functional carboxylic and amino groups, respectively, whereas PLeu represents hydrophobic block. The molecular weight characteristics of the synthesized homopolymers and block copolymers are presented in Tables 2 and 3. Thus, the composition of the labeled copolypeptides can be described as follows: **2**-PLys_119_-*b*-PLeu_141_ and **2**-PGlu_49_-*b*-PLeu_27_.

**Figure 4:**
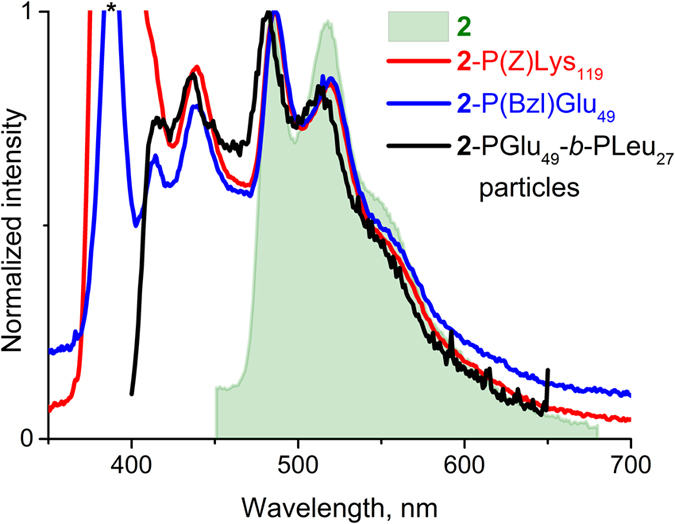
Emission spectra of **2**-P(Z)Lys_119_ and **2**-P(Bzl)Glu_49_ polypeptides (DMSO/chloroform 1/1; λ_exc_ = 385 nm, 22 °C), and 2-PGlu_49_-*b*-PLeu_27_ particles (borate buffer, pH = 9.4; λ_exc_ = 350 nm, 22 °C). Excitation line at 385 nm is marked with an asterisk.

Investigation of the luminescent properties of **2**-P(Z)Lys and **2**-P(Bzl)Glu polypeptides in organic medium (CHCl_3_/DMSO) revealed the presence of emission bands typical for the complex **2** (Fig. 4) along with a weaker high energy emission component (400–450 nm), which can be assigned to organic matrix of polymers.

To estimate the content of the complex **2** in the samples of the synthesized polypeptides the thermogravimetric analyses (see Fig. S6 of Supporting Information) have been carried out. It was found that the content of metal complex with respect to theoretical one equals to 90.1% and 76.9% in **2**-P(Bzl)Glu and **2**-P(Z)Lys, respectively, that points to a rather high degree of the polymers labelling using the procedure suggested.

### Characterization of self-assembled particles

**Figure 5:**
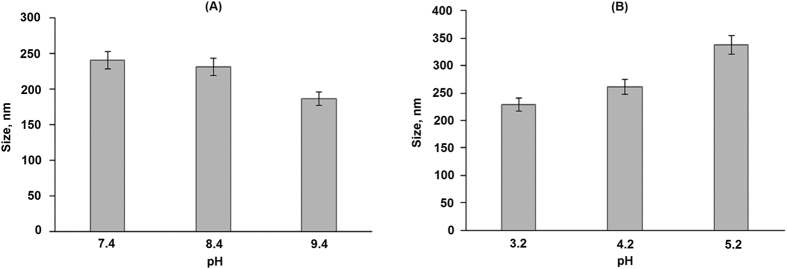
The dependence of 2-PLys_119_-*b*-PLeu_141_ (**A**) and 2-PLys_119_-*b*-PLeu_141_ (**B**) particle size on pH applied for self-assembly.

The synthesized amphiphilic polymers display a tendency to self-assembly in aqueous media that allowed for preparation of polymer particles using the phase inversion method. Since the hydrophilic blocks of the synthesized polypeptides had different functional groups, namely, amino and carboxylic ones, the optimal conditions for the **2**-PLys_119_-*b*-PLeu_141_ and **2**-PGlu_49_-*b*-PLeu_27_ self-assembly were essentially different. In particular, the alkaline medium evidently favors better ionization of carboxylic groups and, consequently, better solubility of **2**-PGlu_49_-*b*-PLeu_27_. In turn, acidic conditions were preferable for the self-assembly of **2**-PLys_119_-*b*-PLeu_141_. The dependence of the particle size on pH used in preparation of ther nanoparticles was established by DLS. The results obtained are presented in Fig. 5.

**Figure 6:**
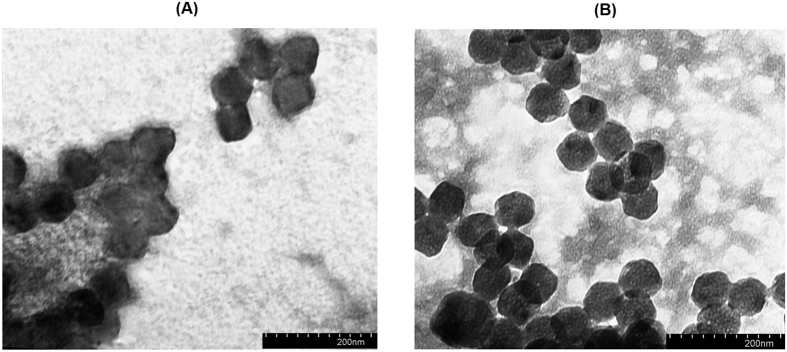
TEM images of 2-PLys_119_-*b*-PLeu_141_ (prepared at buffer, pH 3.2) (**A**) and 2-PLys_119_-*b*-PLeu_141_ (prepared at buffer, pH 9.4) (**B**) nanoparticles.

The average hydrodynamic diameter of the **2**-PGlu_49_-*b*-PLeu_27_ particles was 241 ± 12 at pH 7.4 and 186 ± 24 at pH 9.4 that demonstrated more than 20% decrease in the particle size in alkaline media (Fig. 5A). Under the latter conditions the hydrophilic block (PGlu_49_) of the polymer is getting negatively charged because of acid residues deprotonation. This makes thermodynamically unfavorable self-assembly of the nanoparticles containing large number of like charged polymer chains that results in stabilization of smaller in “molecular weight” and size nanoparticles. A similar trend was observed for the **2**-PLys_119_-*b*-pLeu_141_ particles in acidic media (Fig. 5B) where protonation of Lys amine group prevents formation of large nanoparticles due to repulsion between positively charge polymeric chains. In this case, the hydrodynamic diameter falls in the range 229 ± 14 at pH 3.2, and 338 ± 32 at pH 5.2. Additionally, the formation of the nanoparticles was studied by TEM (Fig. 6), which gives rather narrow particles size distribution. Even smaller size of nanoparticles (ca. 60–80 nm) measured by TEM method can be related to the deflation of water upon drying of particles on a microscope grid before TEM experiments.

Zeta-potential is one of the key characteristics that affects the nanoparticles stability relative to aggregation in colloidal systems. The colloids are considered to be stable if ξ-potential is lower than –30 and higher than +30 mV^48^. Moreover, the high absolute value of zeta potential also favores the increase of drug loading efficiency and simultaneously improve the nanoparticles accumulation in target cells^49^. The particles obtained were characterized with ξ-potential values (in water) equal to −44 and +55 mV for **2**-pGlu_49_-*b*-pLeu_27_ and **2**-pLys_119_-*b*-pLeu_141_, respectively, that is a clear indication of their colloidal stability in aqueous media and high internalization rate. Stability of the particles in physiological solution was controlled by DLS, which showed the absence of aggregation within three weeks in the concentration range 0.005–2.000 mg/ml.

**Figure 7:**
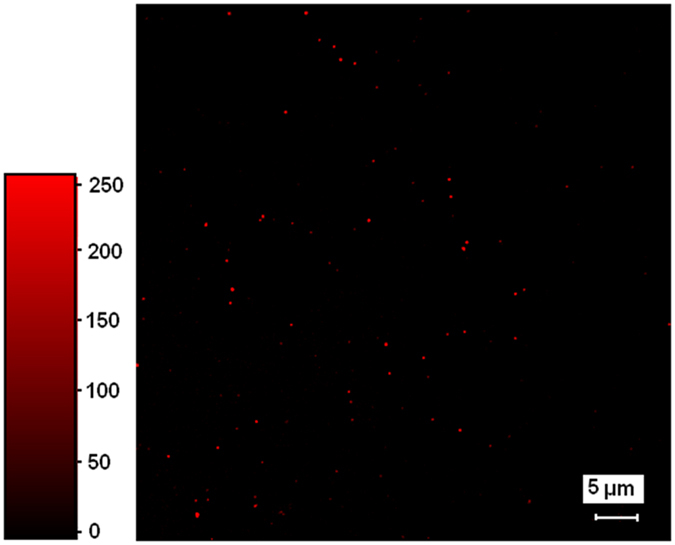
The confocal microscopy of 2-PLys_119_-*b*-PLeu_141_ colloids (buffer, pH 7.4; λ_ex_ = 375–400 nm, λem = 500–530 nm).

The labeled nanoparticles formed from **2**-pGlu_49_-*b*-pLeu_27_ and **2**-pLys_119_-*b*-pLeu_141_ display photoluminescence, see for example, the emission spectrum of **2**-pGlu_49_-*b*-pLeu_27_ particles in buffer solution (pH 9.4), Fig. 4. Obviously, the emission band profile detected for the particles labeled with complex **2** are nearly identical to those measured for soluble label-bearing homopolymer. Additionally, the presence of complex **2** on the surface of polypeptide particles was proved by visualization of nanoobjects by two-photon confocal microscopy (Fig. 7). For better detection, the nanoparticles of ca. 600 nm were prepared by self-assembly of **2**-PLys_119_-*b*-PLeu_141_ polypeptide in Na-phosphate buffer, pH 7.4, and used for analysis.

### Cell experiments: cytotoxicity, hemolysis and cell visualization

**Figure 8:**
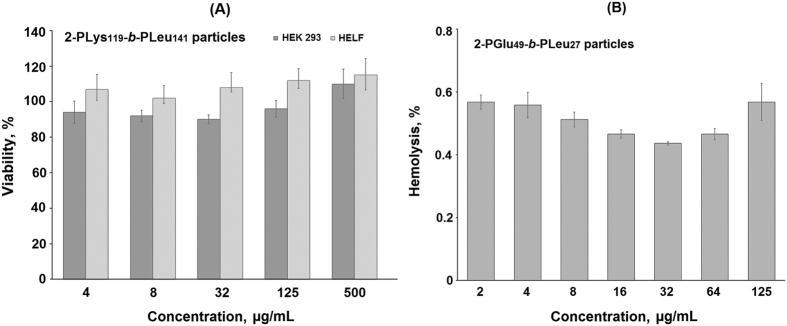
MTT (**A**) and hemolysis (**B**) assays of the luminescent polypeptide nanoparticles.

Recently it was observed that nanoparticles based on PGlu-*b*-PPhe copolymer did not affect cell metabolic activity^31^ that clearly points to the poly(amino acid) nanoparticles biocompatibility. It is reasonable to suppose that the PGlu-*b*-PLeu particles are not cytotoxic as well. However, the particles obtained in this study contain C-terminal Pt-complex that in principle may affect cell viability. To test the biocompatibility of the luminescent nanoparticles, MTT assay using HEK 293 and HELF cell lines was performed. The data shown in Fig. 8A indicate that there are no decrease in cell metabolic activity in the presence of **2**-PLys_119_-*b*-PLeu_141_ during 24 h at the concentrations applied. A similar result was obtained for the **2**-PGlu_49_-*b*-PLeu_27_ particles (Fig. S7 of Supporting Information).

Another important test to prove biocompatibility of the nanoparticles is hemolysis of erythrocytes. The blood biocompatible substances and nanoobjects do not induce the destruction of external membrane of erythrocytes, which causes the release of intracell content. In this case, the toxicity of tested samples is evaluated by estimating the quantity of hemoglobin released. The results of hemolytic test revealed that the hemolysis rate for both **2**-PLys_119_-*b*-PLeu_141_ and **2**-PGlu_49_-*b*-PLeu_27_ particles was lower than 1%. As an example, the dependence of hemolysis rate on concentration of **2**-PGlu_49_-*b*-PLeu_27_ particles is given in Fig. 8B. It is generally accepted that the materials are classified as non-hemolytic if the hemolysis rate is lower than 5%^50^. Thus, the nanoobjects studied can be considered as safe in the tested range of concentrations.

**Figure 9:**
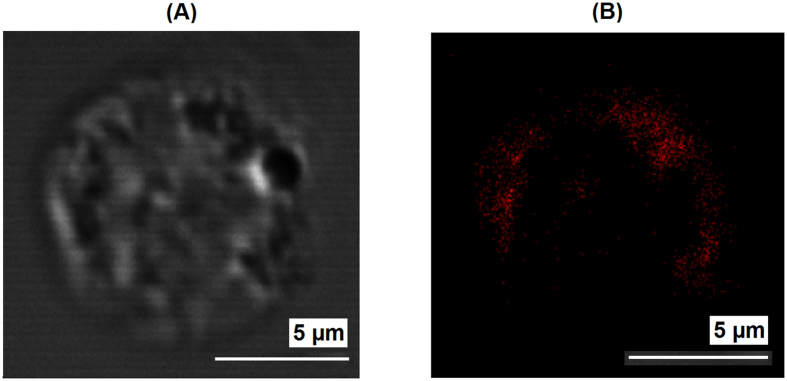
Bright-field (**A**) and confocal fluorescent (**B**); buffer, pH 7.4; λ_ex_ = 375–400 nm, λ_em_ = 500–530 nm) images of U937 cell incubated with **2**-PLys_119_-*b*-PLeu_141_ nanoparticles.

Finally, the luminescent nanoparticles obtained were tested for cell visualization. Fig. 9 and Fig. S8 show the cellular uptake of the **2**-PLys_119_-*b*-PLeu_141_ nanoparticles by U937 cells after 1 hour of co-incubation, which indicates their applicability for luminescent visualization of living cells. The experiment displayed rapid cellular uptake that can be a result of relatively small size of the particles, as well as the positive charge of their surface. The confocal image shows a clear luminescent signal accumulation, which mostly distributed in the cytoplasm at the cell periphery.

## Conclusions

A novel luminescent Pt^II^ complex containing lateral amino group in the ligand environment was synthesized and its photophysical properties were studied. The complex obtained was used as effective ROP initiator to synthesize amphiphilic polypeptides, namely, PLys_119_-*b*-PLeu_141_ and PGlu_49_-*b*-PLeu_27_, with the luminescent label covalently bound to the C-terminal position of the polymer. It was shown that self-assembly of these polypeptides at optimal conditions led to the formation of nanoparticles of 250–350 nm size, which keep intact the luminescence characteristics of the starting platinum complex. The developed luminescent nanoparticles were found to be nontoxic and non-hemolytic in the tested concentration range and can be used for cell visualization with appreciable luminescence intensity in the green-yellow region of visible spectrum. Since such polypeptide-based soft materials are also considered as drug delivery systems, they have a great prospective in the application as duel-function nanoparticles with a combination of diagnostic and therapeutic properties.

## Additional Information

**How to cite this article**: Vlakh, E. G. *et al*. Self-assemble nanoparticles based on polypeptides containing C-terminal luminescent Pt-cysteine complex. *Sci. Rep.* **7**, 41991; doi: 10.1038/srep41991 (2017).

**Publisher's note:** Springer Nature remains neutral with regard to jurisdictional claims in published maps and institutional affiliations.

## Supplementary Material

Supporting Information

## Figures and Tables

**Table 1:** Photophysical properties of **2** in aerated solution at ambient temperature.

Solvent	λ_abs,_ nm (ε, 10^4^ M^−1^cm^−1^)	λ_em_^i^, nm	τ, μs	Q.Y., %
Methanol	260(5.2), 285(2.4), 314(1.4), 410(0.35)	483, 518, ~550	0.039	<1
Dichloromethane	260(1.4), 285(1.1), 311(0.6), 415(0.13)	484, 518, ~550	0.294	6.3
Acetone	—	483, 518, ~551	0.290	6.0

^i^λ_exit_ = 380 nm

**Table 2:** Molecular weight characteristics of labeled homopolypeptides.

Sample	*Mw*	*Mn*	*Mw/Mn*	*n*
**2**-P(Z)Lys	44800	32000	1.4	119
**2**-P(Bzl)Glu	12500	11500	1.1	49

**Table 3:** Molecular weight characteristics of labeled amphiphilic polypeptides.

Sample	Hydrophilic block		Hydrophobic block		Copolymer
	*Mn*	*n*	*Mn*	*m*	*Mn*
**2**-PLys_*n*_*-b-*PLeu_*m*_	16200	119	16000	141	32200
**2**-PGlu_*n*_*-b-*PLeu_m_	7200	49	3150	27	10350
