# Supplementary material for: Self-assemble nanoparticles based on polypeptides containing C-terminal luminescent Pt-cysteine complex
Source: Sci Rep. 2017 Feb 3;7:41991. doi: 10.1038/srep41991 (PMC5290470; doi:10.1038/srep41991)
Supplement: Supporting Information [file srep41991-s1.pdf]

Supporting Information

Self-assemble nanoparticles based on polypeptides containing C-terminal luminescent Pt-cysteine complex

E.G. Vlakh, E.V. Grachova, D.D. Zhukovsky, A.V. Hubina, J.R. Shakirova, A.S. Mikhailova, V.V. Sharoyko, S.P. Tunik*, T.B. Tennikova*

St. Petersburg State University, Institute of Chemistry, Universitesky pr. 26, 198504 St. Petersburg, Russia

* Correspondence: sergey.tunik@spbu.ru; tennikova@mail.ru

Figure S1. ^{1}H NMR spectrum of **1** ***trans*** (acetone-d_6, ambient temperature).

Figure S2. ^{1}H NMR spectrum of **1** ***cis*** (acetone-d_6, ambient temperature).

Figure S3. ^{1}H NMR spectrum of **2** (methanol-d_4, ambient temperature).

Figure S4. ESI^{+} MS spectrum of **1**.

Figure S5. ESI^{+} MS spectrum of **2**.

Figure S6. Thermogram of **2**-P(Bzl)Glu and Pt content calculation.

Figure S7. MTT assay of **2**-PGlu-*b*-PLeu particles.

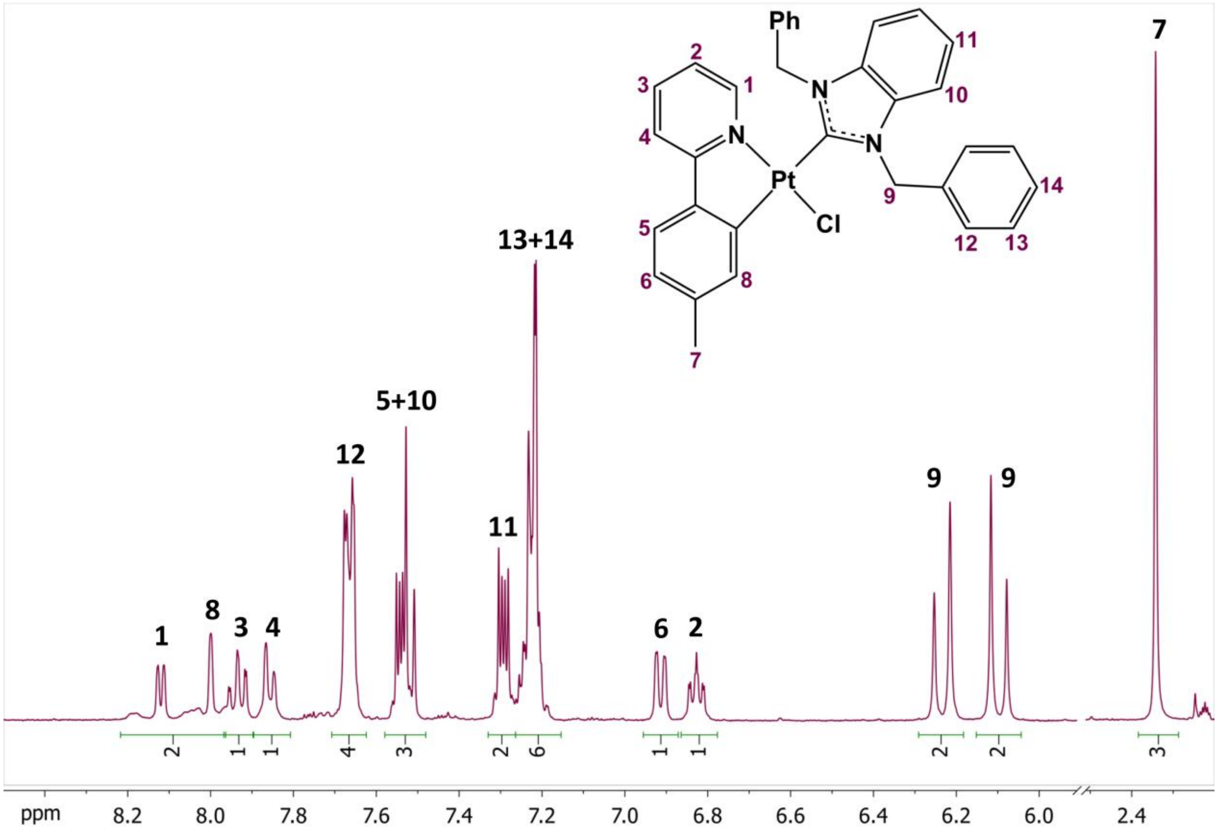

Figure S1. ^{1}H NMR spectrum of **1** ***trans*** (acetone-d_6, ambient temperature).

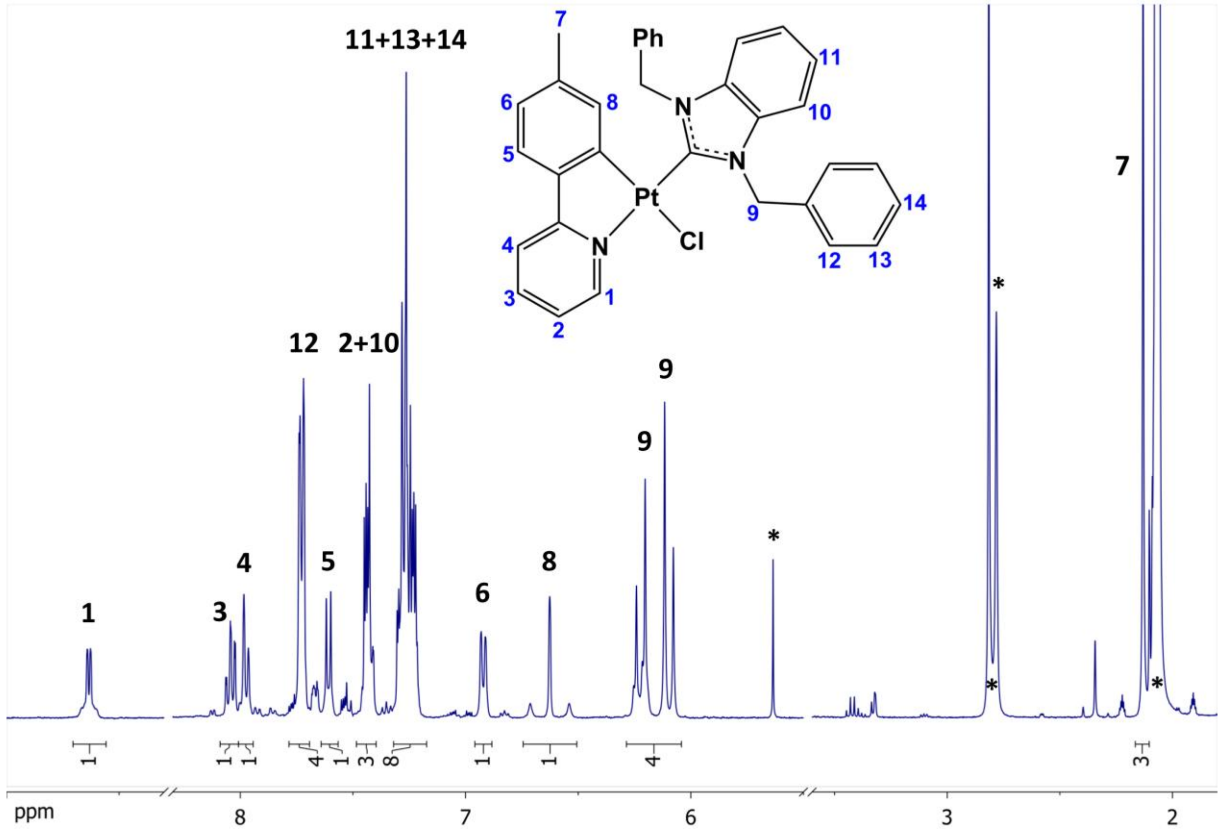

Figure S2. ^{1}H NMR spectrum of **1** ***cis*** (acetone-d_6, ambient temperature).

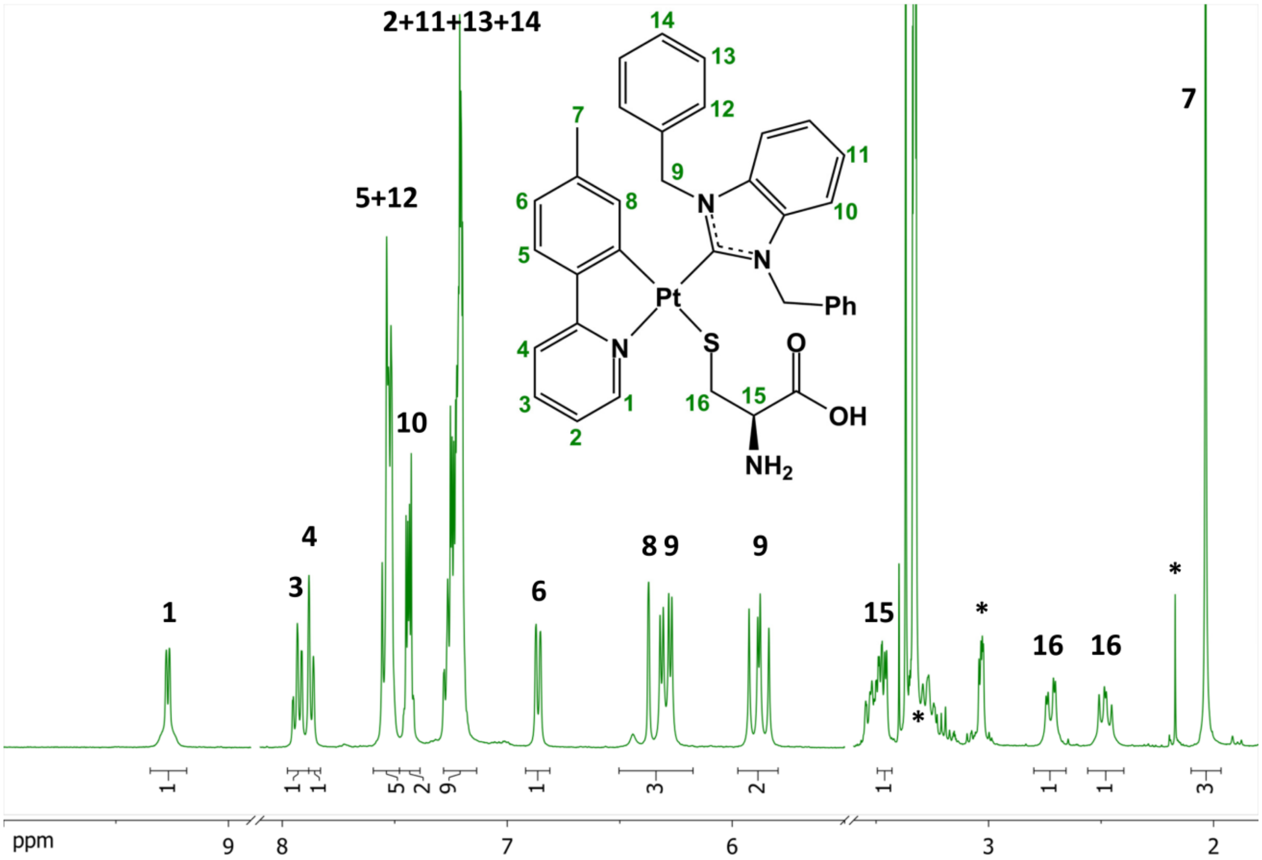

Figure S3. ^{1}H NMR spectrum of **2** (methanol-d$_4$, ambient temperature).

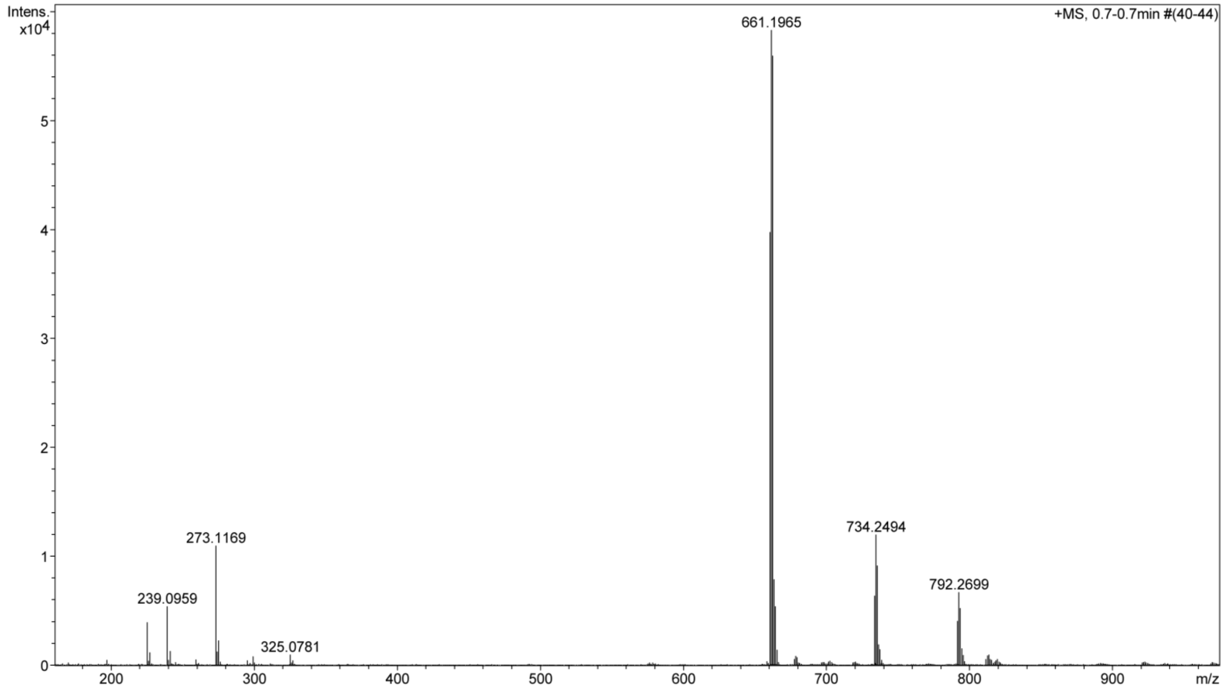

Figure S4. ESI$^+$ MS spectrum of **1**.

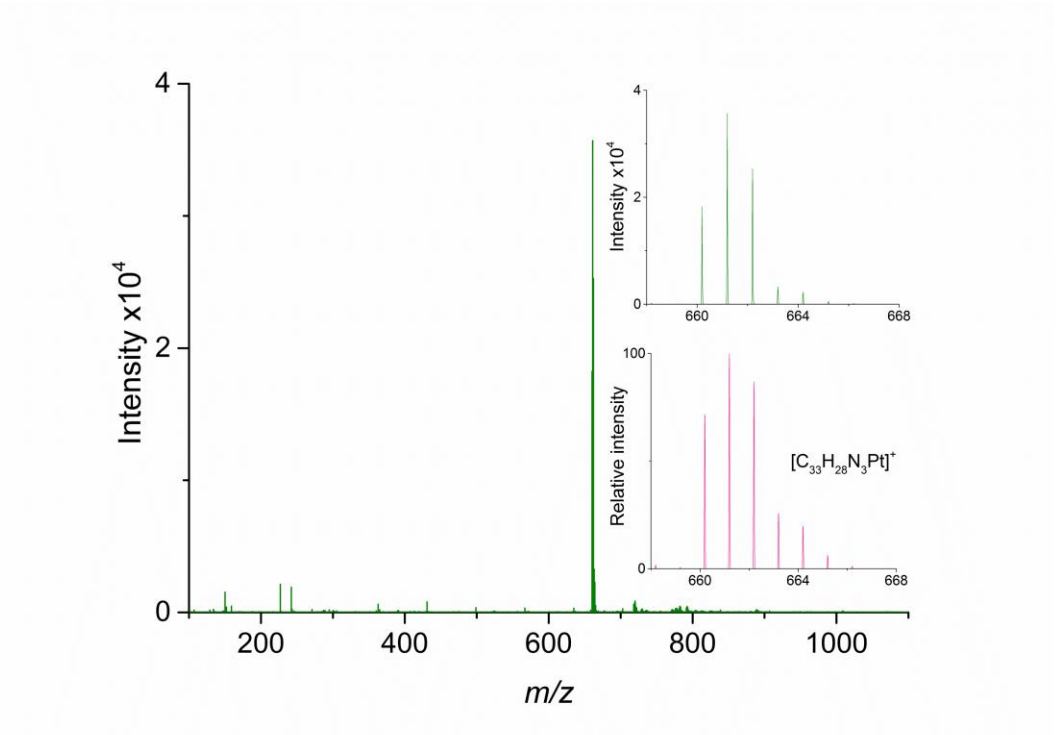

Figure S5. ESI$^+$ MS spectrum of **2** (green − experimental spectrum, pink − simulated signal with *m/z* 661.19 [M − Cys]$^+$).

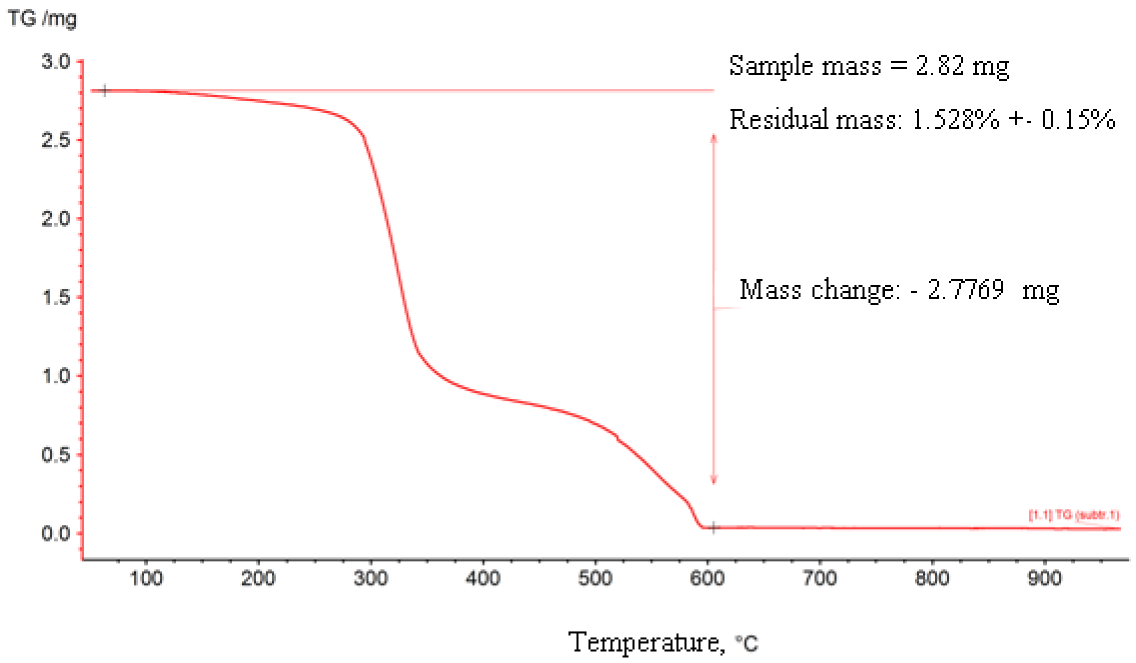

Figure S6. Thermogram of **2**-P(Bzl)Glu and Pt content calculation *vide infra*.

The Pt content in the samples was calculated as follows:

$$m(Pt)_{theoretical} = \frac{M\ (Pt) \times m(polymer)}{Mn(polymer)};$$

$$\eta = m\ (Pt)_{TGA} / m\ (Pt)_{theoretical} \times 100\%$$

Thus, for **2**-P(Bzl)Glu:

$m\ (Pt)_{TGA} = 0.0431$ mg

$m\ (Pt)_{theoretical} = (195 \times 2.82)/11500 = 0.04782$ mg

$\eta = 0.0431/0.04782 \times 100\% =$ **90.1 %**

According to TGA analysis, the mass change for **2**-P(Z)Lys was equal to 0.47%, that corresponds to $m\ (Pt)_{TGA} = 0.02462$ mg. Taking into account that $m\ (Pt)_{theoretical}$ was 0.03199 mg, η was calculated to be **76.9%.**

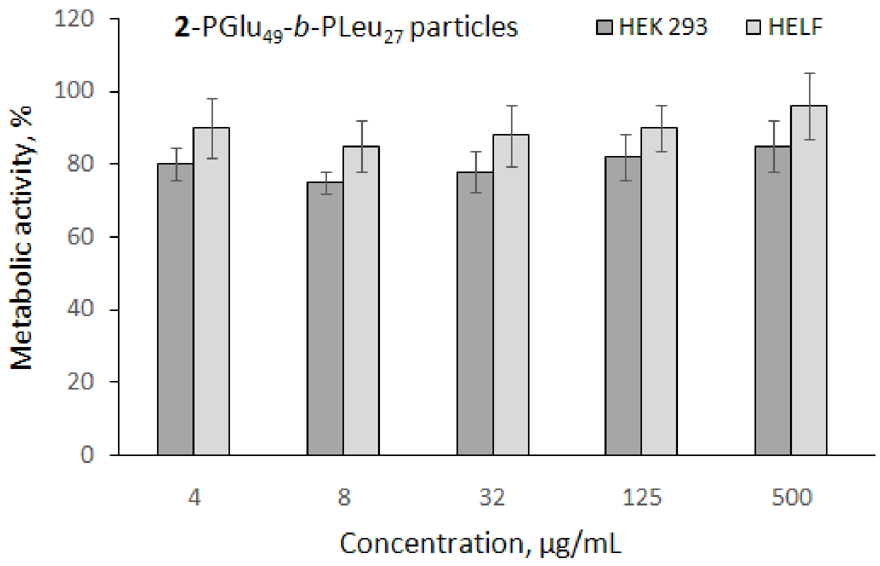

Figure S7. MTT assay of **2**-PGlu-*b*-PLeu particles.

(A)

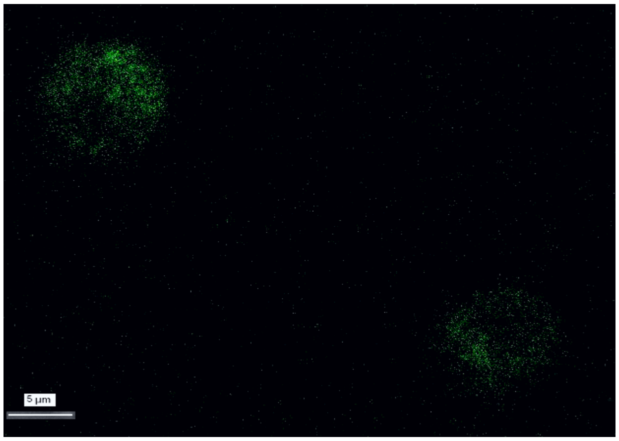

(B)

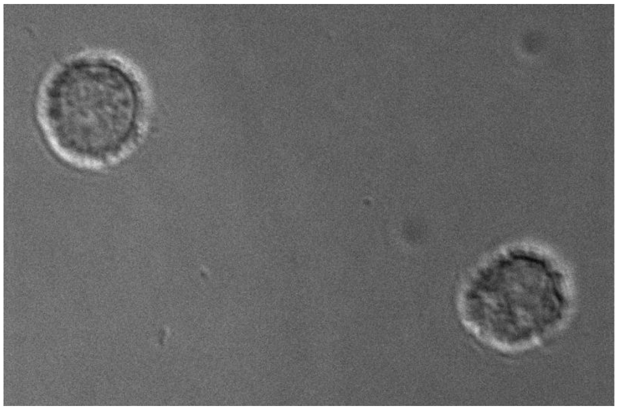

Figure S8. Broad area confocal images of U937 cells incubated with **2**-PLys$_{119}$-*b*-PLeu$_{141}$ nanoparticles: (A) fluorescent (buffer, pH 7.4; λ_{ex} = 375-400 nm, λ_{em} = 500-530 nm) and bright-field (B).
